# A Comparative Study on Inhibition of Total Astragalus Saponins and Astragaloside IV on TNFR1-Mediated Signaling Pathways in Arterial Endothelial Cells

**DOI:** 10.1371/journal.pone.0101504

**Published:** 2014-07-03

**Authors:** Qin-she Liu, Hai-fang Wang, An-ke Sun, Xue-ping Huo, Jin-lian Liu, Shu-hui Ma, Ning Peng, Jun Hu

**Affiliations:** 1 Department of Public Health, Medical School of Xi’an JiaoTong University, Xi’an, China; 2 Laboratory Center of Shaanxi Province People’s Hospital, Xi’an, China; 3 Shaanxi Province Institute of Chinese Medicine and Medicinal Herbs, Xi’an, China; 4 Department of Otolaryngology, General Hospital of Shenyang Military Command, Shenyang, China; 5 Department of Clinical Traditional Chinese Medicine-Western Medicine, Medical School of Xi’an JiaoTong University, Xi’an, China; IISER-TVM, India

## Abstract

**Background:**

Both total astragalus saponins (AST) and it’s main component astragaloside IV (ASIV) have been used in China as cardiovascular protective medicines. However, the anti-inflammatory activities that are beneficial for cardiovascular health have never been compared directly and the molecular mechanisms remain unresolved. This study was conducted to compare the inhibitory effects of these drugs on TNFα-induced cell responses, related signaling pathways, and the underlying mechanisms in mouse arterial endothelial cells.

**Methodology/Principal Findings:**

Real-time qRT-PCR was performed to determine the expression of cell adhesion molecule (CAM) genes. Immunofluorescent staining was used to detect the nuclear translocation of transcription factor NF-κB-p65. Western Blot analysis was used to identify TNFα-induced NF-κB-p65 phosphorylation, IκBα degradation, and caspase-3 cleavage. Cell surface proteins were isolated and TNFα receptor-1(TNFR1) expression was determined. The results suggest that both AST and ASIV attenuate TNFα-induced up-regulation of CAMs mRNA and upstream nuclear translocation and phosphorylation of NF-κB-p65. However, TNFR1-mediated IκBα degradation, cleavage of caspase-3 and apoptosis were inhibited only by AST. These differences in the actions of AST and ASIV could be explained by the presence of other components in AST, such as ASII and ASIII, which also had an inhibitory effect on TNFR1-induced IκBα degradation. Moreover, AST, but not ASIV, was able to reduce TNFR1 protein level on the cell surface. Furthermore, mechanistic investigation demonstrated that TNFR1-mediated IκBα degradation was reversed by the use of TAPI-0, an inhibitor of TNFα converting enzyme (TACE), suggesting the involvement of TACE in the modulation of surface TNFR1 level by AST.

**Conclusion:**

ASIV was not a better inhibitor than AST, at least on the inhibition of TNFα-induced inflammatory responses and TNFR1-mediated signaling pathways in AECs. The inhibitory effect of AST was caused by the reduction of cell surface TNFR1 level, and TACE could be involved in this action.

## Introduction

Radix Astragali (Astragalus) taken from the medicinal plant Astragalus membranaceus has been used in traditional Chinese medicine (TCM) for thousands of years. It is used primarily to protect and boost the body’s immune system, but is also widely prescribed to treat cardiovascular disorders. However, the mechanisms of astragalus’s actions remain largely a mystery because it is usually taken as whole herb preparation. The multiple active components contained in astragalus may work together to fight diseases, which also is one of the fundamental instructive principles of TCM. Over the past several decades, much progress has been made in the separation and analysis of the active pharmaceutical ingredients in herbal medicines including astragalus [1], [2], [3]. Saponins are surface-active steroids or triterpenoid glycosides found in a large number of herbal plants and are considered to be responsible for the pharmacological activity of many Chinese medicines. Studies have illustrated the beneficial effects of saponins on blood cholesterol levels, cancer, bone health and stimulation of the immune system [4]. Increasing attention has been paid to the anti-oxidative and anti-inflammatory effects of saponins contained in different medicinal plants which may be beneficial to the cardiovascular system [5], [6].

One of the main bioactive constituents in astrgalus are astrgalosides - cycloartane-type triterpenoid saponins [2], [3]. Moreover, a variety of structurally distinct substances (e.g., astragalosides I-VII) have been identified in total saponins of astragalus membranaceus (AST) among which ASIV is considered the primary active component [1], [2]. A number of in vivo experiments have illustrated the cardiovascular protective effects of AST [7], [8] and ASIV [9], [10], which may be partially due to their anti-oxidative, anti-inflammatory and anti-apoptotic activities that are endothelium-protective under pathophysiological conditions. Based on their effectiveness and low toxicity as demonstrated in animal studies, both AST and ASIV have been used as complementary medicines for the treatment of cardiovascular diseases in China. However, many of the previous mechanistic studies were performed with ASIV [11], [12], and the pharmacological actions of ASIV and AST have never been compared directly. The preparation procedure of AST is more convenient and costs much less than that of ASIV, and ASIV has a low solubility in water which makes its’ clinical application more difficult. A direct comparison of these two drugs is required to provide useful information for future drug development and also clinical drug selection.

Vascular endothelial cell (VEC) dysfunction has been widely accepted to play an important role in a variety of cardiovascular diseases (CVDs), including atherosclerosis, hypertension and diabetic vascular diseases. Tumor necrosis factor-α (TNFα), is among the most critical pro-inflammatory cytokines in human body and plays an important role in the modulation of VEC function through activation of multiple intracellular signaling pathways. TNFα binds and signals through two distinct receptors, TNFR1 (TNF Receptor type-1) and TNFR2 (TNF Receptor type-2). TNFR1 is expressed in all human tissues and is the major signaling receptor for TNFα. Through binding to and activating TNFR1 on VEC surface, TNFα can promote the activation of transcription factor nuclear factor- κB (NF-κB) and p-38 and ERK1/2 MAPKs (Mitogen-activated protein kinases) signaling pathways, thereby up-regulating the expression of cell adhesion molecules (CAMs), including intracellular adhesion molecule-1 (ICAM-1) and E-selectin, which are involved in the processes of leukocyte attachment and transmigration. TNFα may also stimulate caspase-3 activation and apoptotic cell death in VECs through activation of extrinsic pro-apoptotic signaling pathways [13], which, in turn contributes to the rupture of atherosclerotic plaques [14]. Many cardiovascular agents act, at least partly, through reducing TNFα-stimulated inflammatory responses of VECs [15], [16].

In the present study, the inhibitory effects of AST and ASIV on the TNFα-induced expression of CAMs were measured in mouse kidney arterial ECs. The influence of these two drugs on TNFR1-mediated activation of NF-κB, MAPKs and pro-apoptotic signaling pathways were determined and the underlying mechanisms were investigated and compared. Surprisingly, our results demonstrated that, although they both were able to attenuate TNFα-induced expression of ICAM-1 and E-selectin, AST and ASIV acted in different ways and involve different mechanisms, suggesting that there might be some currently unidentified difference in their clinical indications.

## Materials and Methods

### Drugs, Reagents and kits

Chemically pure astragaloside total (AST) and astragaloside IV (ASIV) were purchased from the National Institute for the Control of Pharmaceutical and Biological Products (Beijing, China) and their purity is greater than 98%. Stock solutions of AST and ASIV were made in DMSO. The final concentration of DMSO added to cells was never greater than 0.1%. Recombinant mouse TNFα was obtained from Calbiochem (San Diego, CA, USA). Cycloheximide (CHX) was purchased from Sigma (St. Louis, MO, USA). TAPI-0 (TNF-α processing inhibitor-0), a synthetic inhibitor of TNFα converting enzyme (TACE), was purchased from Enzo Life Sciences Inc (Farmingdale, NY, USA). RPMI 1640, glutamine, penicillin/streptomycin, and HEPES were purchased from Clontech Laboratories (Palo Alto, CA, USA). Fetal calf serum (FCS) was purchased from Hyclone Laboratories (Logen, UT, USA). Total RNA isolation kits, reverse transcription (RT) kits and SYBR Green PCR Master MIX were all purchased from Takara Bio Inc. (Dalian, China). Cell Surface Protein Isolation Kits were purchased from Pierce (Rockford, IL, USA).

### Cell culture & TNFα Treatment

Primary mouse kidney arterial endothelial cells (AECs) were provided by Bioleaf Biotech Co. Ltd, (Shanghai, China) and grown in RPMI-1640 media supplemented with 15% FCS, 100 U/mL penicillin and 100 µg/mL streptomycin. Immunofluorescent staining with anti-von Willebrand Factor VIII (VWF) antibody was used to confirm that we had endothelial cells. For the induction of IκBα degradation and expression of CAM genes, cells were treated with 30 ng/mL of TNFα for 30 minutes (min) and 6 hours (h), respectively. For the induction of apoptosis, cells were treated with 30 ng/mL of TNFα with 10 µg/mL of cyclohexamide (CHX) for 8 h. Apoptosis was evaluated by observing the cellular morphologic changes under light microscope and Western blot analysis of caspase-3. Cells were used through passage five.

### Immunofluorescent staining

AECs were cultured for 2 days on glass coverslips in 6-well plates. After stimulation, the cells were washed with PBS three times and then fixed in 4% paraformaldehyde for 30 min and permeabilized in 0.2% Triton X-100 for 5 min at room temperature. Cells were stained with a polyclonal antibody against NF-κB p65 (1∶100) (Santa Cruz Biotechnology, Santa Cruz, CA, USA) followed by 1∶500 of FITC-conjugated secondary antibody (Sigma). Images were recorded using an Olympus digital camera attached to a Nikon Diaphot 200 inverted fluorescent microscope. All experiments were performed at least three times. Representative images are displayed in the figures. Rabbit polyclonal antibody against VWF was purchased from Santa Cruz and used for the identification of AECs.

### SYBR Green real-time quantitative PCR

Approximately 2×10^6^ cells/sample were prepared for extraction of total RNA. mRNA expression levels for ICAM-1 and E-selectin in cells were quantified by SYBR Green two-step, real-time RT-PCR. β-actin was used as an internal standard. 1 µg of total RNA from each sample was used for reverse transcription with oligo dT and Superscript II to generate first-strand cDNA. PCR reactions were prepared using SYBR Green PCR Master Mix. The primer sequences were as follows: ICAM-1, forward: 5′- GTG GGT CGA AGG TGG TTC TT-3′, and reverse: 5′- AGG CCT GGC ATT TCA GAG TC-3′; E-selectin, forward: 5′- CCT AAG GGA TCC AAC GCC AG-3′, and reverse: 5′- GAG CTC ACT GGA GGC ATT GT-3′; β-actin, forward: 5′- TAG GCG GAC TGT TAC TGA GC-3′, and reverse: 5′-TGC TCC AAC CAA CTG CTG TC-3′. The PCR reaction consisted of 1 min denaturation step at 95°C followed by 40 cycles of 15 sec at 95°C and 30 sec at 56°C on an Eco Real-Time PCR System (Illumina). All samples were run in triplicates.

### Western blot analysis and antibodies

After drug treatment, cells extracts were collected in 2×SDS sample buffer and protein concentrations were determined with Amido Black Protein Assay. Equal amounts of total protein lysate (20 µg) were loaded onto SDS-PAGE gels for separation and transferred to nitrocellulose membrane for immunoblotting. The following primary antibodies were used: ph-NF-κB, IκBα, and cleaved caspase-3 (Cell Signaling Technology, Danvers, MA, USA), β-actin (CoWin Bioscience, Beijing, China), TNFR1 (Abcam, Cambridge, MA, USA), cadherin-11 (Life Technologies, Carlsbad, CA, USA). The appropriate anti-mouse Ig and peroxidase-conjugated anti-rabbit Ig secondary antibodies (CoWin Bioscience, Beijing, China) were used. The signals were detected and quantified using a Fluorchem FC2 Luminescent Imager (Alpha Innotech).

### Cell surface TNFR1 protein analysis

AECs grown in 10 cm dishes were washed three times with ice-cold PBS and incubated with 0.25 mg/mL of EZ link NHS-Sulfo-SS-biotin in PBS for 30 min at 4°C. The reaction was quenched and the cells were then lysed in 600 µL of cell surface protein lysis buffer (50 mM Tris–HCl, 500 mM NaCl, 1 mM EDTA/EGTA, 0.5% SDS, 1% Triton X-100, and 0.1% NP-40, pH 7.4) containing fresh protease inhibitors (1 µM pepstatin A, 250 µM phenylmethylsulfonyl fluoride, 1 µg/mL leupeptin, and 1 µg/mL aprotinin). The lysates were then sonicated for 40 s on ice and centrifuged at 10,000 g for 15 min at 4°C. The supernatants were incubated with Neutravidin beads (100 µL) for 2 h at 4°C. Beads were washed three times with fresh lysis buffer, and bound proteins were eluted with 60 µL of 2× Western blot sample buffer for 6 min at 100°C. Aliquots from total cell lysates (20 µg) and Neutravidin-bound samples (30 µL) were analyzed by Western blot analysis. Biotinylated TNFR1 protein (representing the surface TNFR1) was normalized using levels of cadherin-11 on the cell surface, and values were averaged across three experiments.

### Statistical analysis

Data were analyzed with software of SPSS14.0. Statistical significance was assessed by one-tailed t-tests or analysis of variance as indicated, and a P-value of <0.05 was interpreted as statistically significant. Experiments were performed in triplicate.

## Results

### Immunofluorescence identification of AECs

**Figure 1:**
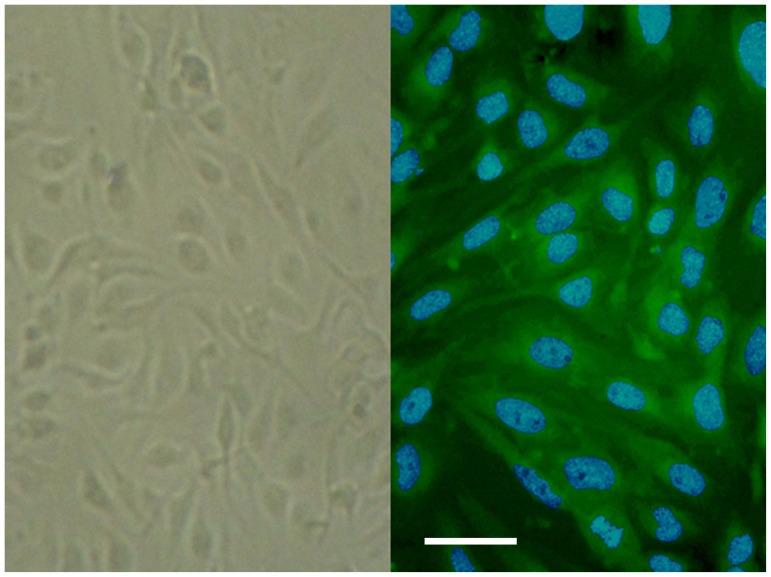
Mouse arterial endothelial cells (mAECs). Cells were observed under light microscope (Left), or after fluorescence staining with vWF (Right). Cultured cells were fixed and treated with rabbit anti-mouse vWF antibody followed by FITC-conjugated anti-rabbit immunoglobulin G. vWF antigen was strongly identified in the cytoplasm (green). The cell nuclei were stained by Hoescht 33258 (blue). Scale bar: 20 µm.

The cells used in this study were provided by Bioleaf Biotech Co. Ltd, as primary endothelial cells isolated from mouse kidney arteries (Arterial Endothelial Cells, AECs). Observed under the light microscopy, the cell bodies were in various shapes - round, oval, triangular or irregular. The number of cell processes varied from two to six, and some processes were bifurcated. The monolayer of cells was irregular in arrangement (Figure 1, Left). von Willebrand factor (VWF) is a glycoprotein routinely used to identify endothelial cells isolated from vascular tissues. To determine the expression of VWF, we performed indirect immunofluorescent staining. As shown in Figure 1, VWF expression was detected as green signals in the cytoplasm of almost all cells, indicating that our cells were indeed endothelial cells (Figure 1, Right).

### AST and ASIV decreased TNFα-induced up-regulation of CAMs mRNA

**Figure 2:**
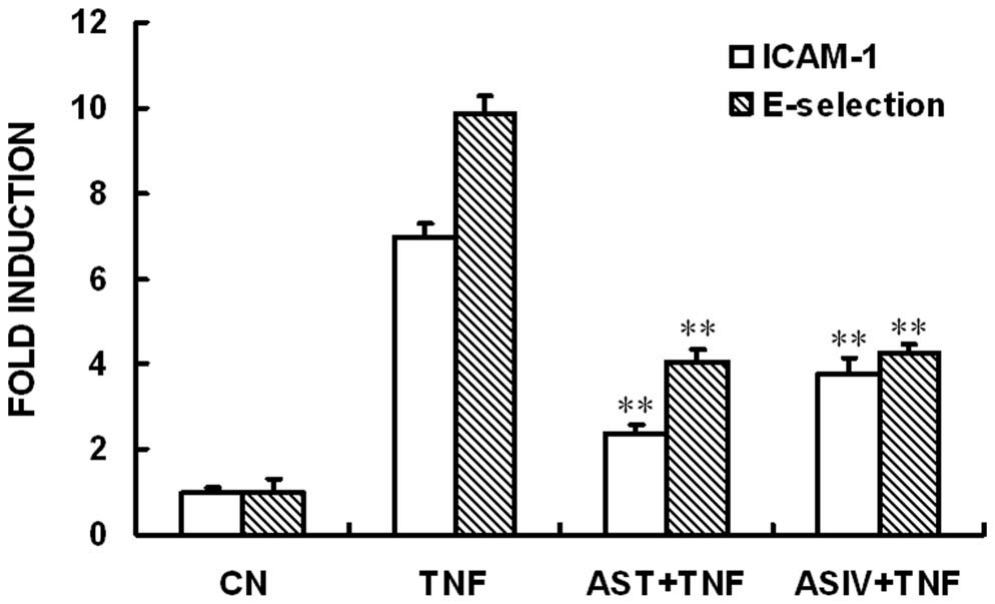
Effects of AST and ASIV on TNFα-induced up-regulation of ICAM-1 and E-selectin mRNAs. Confluent mAECs were pre-incubated with 250 µg/mL of AST or ASIV for 2 h, then washed and incubated for 6 h with 30 ng/mL TNFα. Cells were then collected and analyzed for ICAM-1 expression using real-time qPCR. Data are representative of three independent experiments with determinations made in triplicate and shown as mean values+S.D. **P<0.01 compared with cells treated with TNFα alone. CN: Control.

Pro-inflammatory cytokines are able to induce the expression of an array of cell adhesion molecules (CAMs), including ICAM-1 and E-selectin, thus playing an important role in certain cellular responses such as leukocyte attachment to the vascular wall and transmigration of leukocytes into regions of inflammation, which have been reported to be involved in vascular inflammation and related diseases. ASIV was reported to decrease LPS- or TNFα-induced expression of VCAM-1 (vascular cell adhesion molecule 1) and E-selectin in human umbilical vein endothelial cells (HUVECs) [11]. Here, by performing real-time qPCR assays, we showed evidence that mRNA levels of ICAM-1 and E-selectin in AECs increased remarkably after exposure of the cells to TNFα for six hours. This induction was significantly reduced when the cells were pre-incubated with 250 µg/mL of AST or ASIV for two hours prior to TNFα stimulation (Figure 2). AST or ASIV alone did not affect the cell viability and cellular expression levels of E-selectin or ICAM-1 (data not shown). The concentrations of AST and ASIV employed in this experiment were based on previous in vitro studies [11]. Given that our main purpose was to dissect the differences in the mechanisms underlying the anti-inflammatory effects, we did not measure the concentration-response curves of these two drugs.

### AST and ASIV suppressed TNFα-induced NF-κB p65 nuclear translocation and activation, while only AST inhibited TNFα-induced IκBα degradation

**Figure 3:**
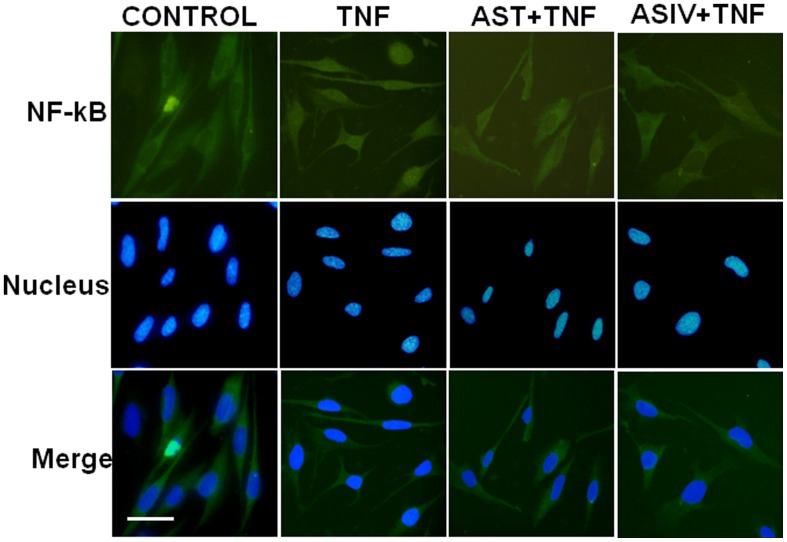
Effects of AST and ASIV on TNF-induced NF-κB-p65 translocation. Confluent mAECs were pre-incubated with 250 µg/mL of AST or ASIV for 2 h, then washed and incubated for 50 min with 30 ng/mL of TNFα. The cells were then fixed and stained for NF-κB-p65 signal. Scale bar: 20 µm.

**Figure 4:**
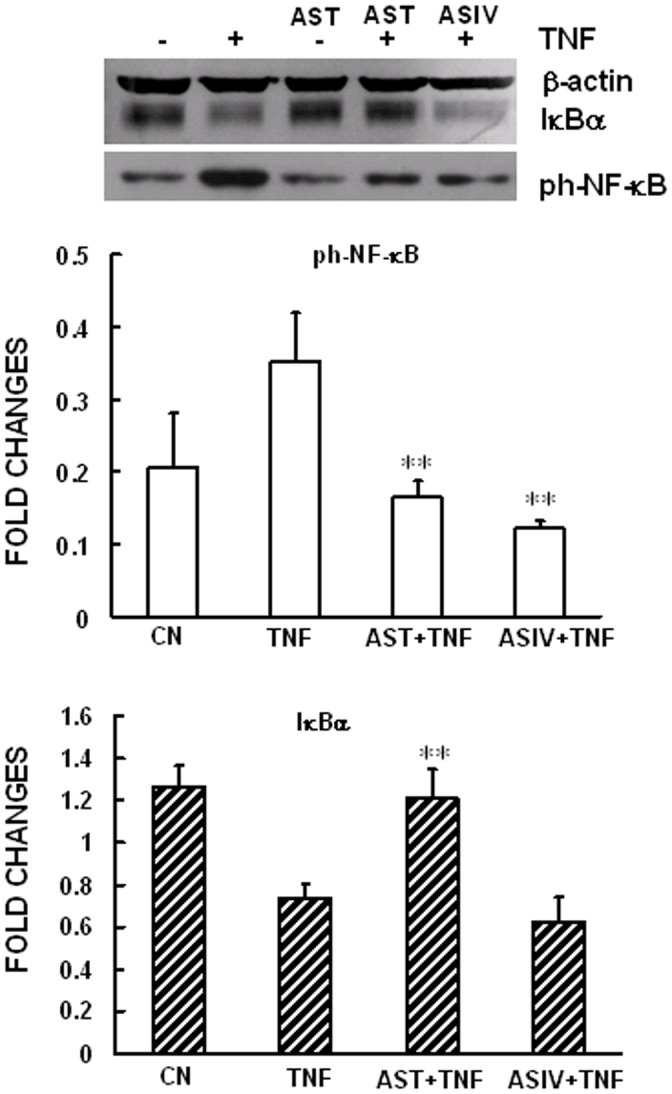
Effects of AST and ASIV on TNF-induced NF-κB-p65 phosphorylation and IκBα degradation. Confluent mAECs were pre-incubated with 250 µg/mL of AST or ASIV for 2 h, then washed and incubated for 30 min with 30 ng/mL TNFα. Cells were then collected and analyzed for expression of phosphorylated NF-kB-p65 and IκBα by Western blott analysis. Data are representative of three independent experiments with determinations made in triplicate and shown as mean values+S.D. **P<0.01 compared with cells treated with TNFα alone. CN: Control.

**Figure 5:**
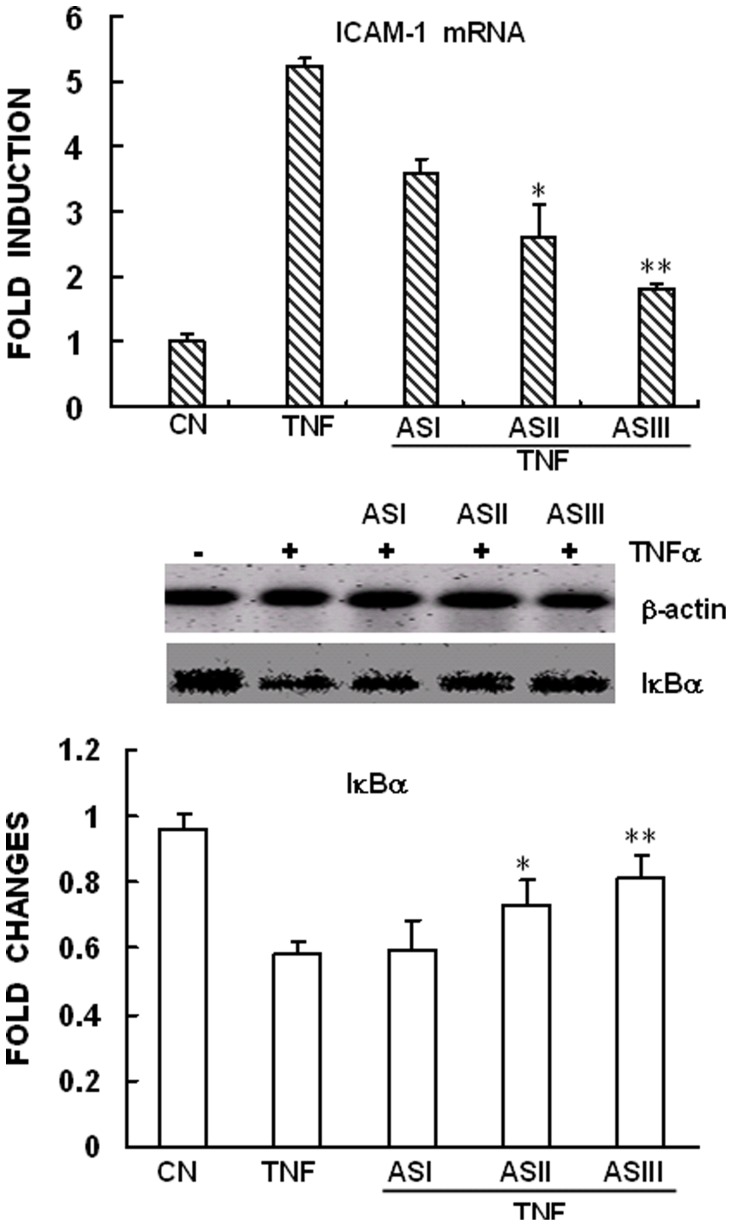
Effects of ASI, ASII and ASIII on TNF-induced ICAM-1 expression and IκBα degradation. Confluent mAECs were pre-incubated with 250 µg/mL ASI, ASII or ASIII for 2 h, then washed and incubated for 6 h or 30 min with 30 ng/mL TNFα. Cells were then collected and analyzed for the expression of ICAM-1 mRNA or IκBα by performing real-time qPCR and Western blot analysis, respectively. Data were representative of three independent experiments with determinations made in triplicate and shown as mean values+S.D. * P<0.05, **P<0.01 compared with cells treated with TNFα. CN: Control.

In quiescent cells, the transcription factor NF-κB is sequestered in the cytoplasm bound to its inhibitors, IκBα (Inhibitor of κB α) thus preventing entry into the nucleus. Following cellular stimulation by TNFα, IκBα is rapidly degraded, allowing for the nuclear retention and activation of NF-κB p65 subunit (NF-κB-p65) and promotion of downstream gene transcription. In the present study, the appearance of NF-κB-p65 in the nucleus was visualized by immunofluorescent staining and the activity of NF-κB was measured by Western blot analysis of phosphorylated NF-κB-p65. Our results showed that, in accordance with previous work of Zhang et al [15], exposure of the cells to ASIV for 2 h significantly prevented TNFα-induced NF-κB-p65 nuclear translocation and phosphorylation of p65, which is important for its transcriptional activity. Similar inhibitory effects on NF-κB-p65 nuclear translocation and phosphorylation were also observed in cells pre-incubated with AST (Figure 3, 4). This is quite conceivable as ASIV has been accepted as the major component of AST. However, surprisingly, we found that pretreatment of the cells with AST significantly attenuated TNFα-induced degradation of IκBα, the upstream event of NF-κB-p65 activation (Figure 4). In contrast, ASIV did not show any inhibitory effect on TNFα-induced IκBα degradation. Taken together, this suggests that different mechanisms could be involved in the anti-inflammatory actions of these two drugs. Considering that AST is known to be composed of a series of structurally different saponins, our assumption is that the effect of AST could be explained by one or more of these active components. Therefore, we determined the effects of ASI, ASII and ASIII, on TNFα-induced up-regulation of ICAM-1 expression and degradation of IκBα. As shown in Figure 5, TNFα-induced IκBα degradation and ICAM-1 expression were significantly inhibited by ASII and ASIII, but not ASI, providing a strong support for our hypothesis.

### AST, but not ASIV, inhibited TNFα-induced pro-apoptotic signaling pathway

**Figure 6:**
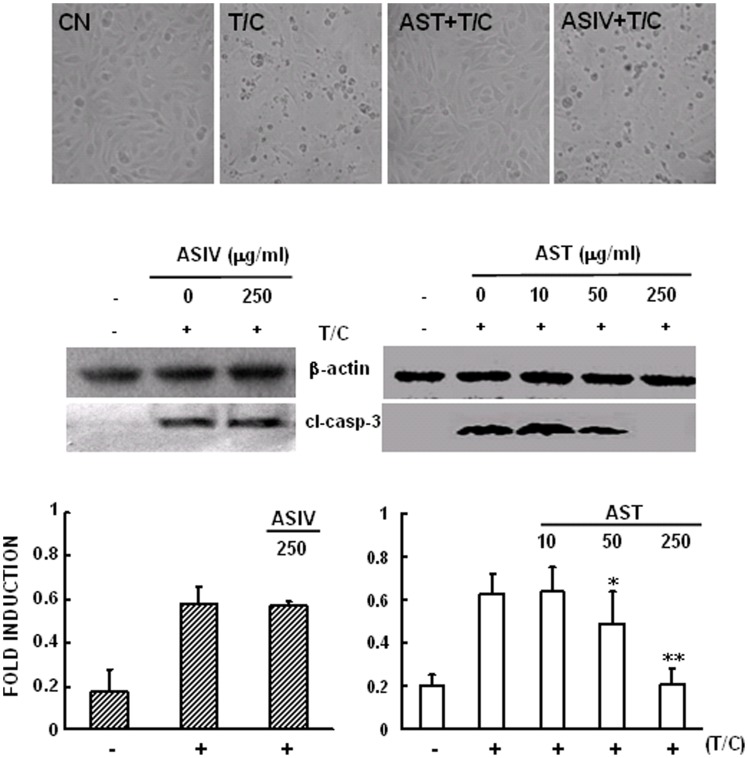
Effects of AST and ASIV on TNF-induced apoptosis. Confluent mAECs were pre-incubated with 250 µg/mL AST or ASIV for 2 h, then washed and incubated for 8 h with 30 ng/mL TNFα+10 µg/mL CHX (T/C). The cells were then photographed, collected and analyzed for the expression of cleaved-caspase 3 by Western blot analysis. Data were representative of three independent experiments with determinations made in triplicate and shown as mean values+S.D. * P<0.05, **P<0.01 compared with cells treated with TNF+CHX. CN: control; T/C: TNF+CHX.

In addition to leading to activation of the IκBα/NF-κB signaling pathway, TNFα is also capable of inducing activation of caspase-3 and apoptosis in VECs. This is mediated by cell surface TNFR1 and can be potentiated by concomitant use of CHX, an inhibitor of protein biosynthesis [13]. TNFα-induced apoptosis in ECs also contributes to the pathogenesis of many cardiovascular diseases [14]. In this study, apoptosis was evaluated by observing the cellular morphologic changes under light microscope and Western blot analysis of caspase-3 cleavage. As shown in Figure 6, significant cellular morphologic changes were observed and cleavage of caspase-3, which represents the processing and activation of caspase-3, was induced by treatment of cells with TNFα plus CHX for 8 h. Pre-exposure of the cells to AST for 2 h inhibited cleavage of caspase-3 in a concentration-dependent manner. TNFα-induced morphological changes were negligible and cleavage of caspase-3 was almost completely abolished by addition of 250 µg/mL AST for two hours. We also investigated the time-dependence of the anti-apoptotic effect of AST. The result showed that a one hour pre-incubation of cells with AST 250 µg/mL was sufficient for its anti-apoptotic effect (data not shown). In contrast, TNFα-induced apoptosis was not affected by exposure of the cells to the same concentration of ASIV. Taken together, these results strongly suggest that, although having identical inhibitory effect on TNFα-induced expression of CAMs genes, AST and ASIV acted in different modes and distinct mechanisms could be involved in their pharmacological actions, which might be caused by other saponin components, such as ASII and ASIII, contained in AST.

### AST, but not ASIV, reduced cell surface level of TNFR1 protein

**Figure 7:**
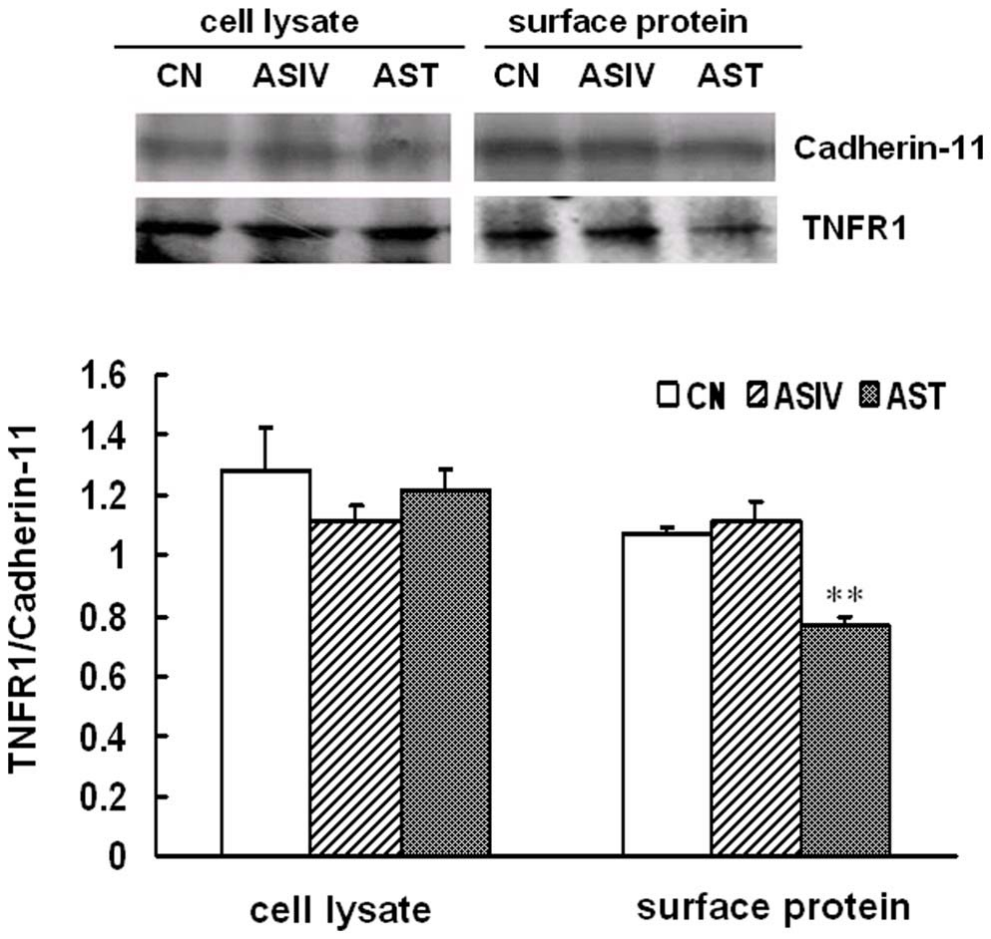
AST, but not ASIV, reduced surface expression of TNFR1 protein. Confluent mAECs were incubated with 250 µg/mL AST or ASIV for 2 h, then cell surface proteins were isolated and TNFR1 expressed on the cell surface was measured by Western blot analysis. Data were representative of three independent experiments with determinations made in triplicate and shown as mean values+S.D. **P<0.01 compared with cells treated with control group. CN: control.

Based on its inhibitory effects on both TNFR1-mediated IκBα/NF-κB and pro-apoptotic signaling pathways, we assumed that AST could act through preventing the initiation of TNFα induced TNFR1-mediated signaling. To test this directly, we investigated the effect of AST and ASIV on upstream initiation of TNFR1 signaling by measuring cell surface expression level of TNFR1. Total cell surface proteins were isolated and the amount of total TNFR1 in cell lysates and the amount of cell surface TNFR1 were analyzed by Western blot using cadherin-11 as an internal reference. As shown in Figure 7, neither AST nor ASIV induced significant changes in the total levels of TNFR1 in cell lysates, however, the amount of cell surface TNFR1 was significantly reduced (35%) by 250 µg/mL of AST, but not ASIV, implying that modulation of cell surface TNFR1 might be involved in the pharmacological actions of AST.

### TAPI-0 prevented the inhibitory effect of AST on TNFR1-mediated IκBα signaling

**Figure 8:**
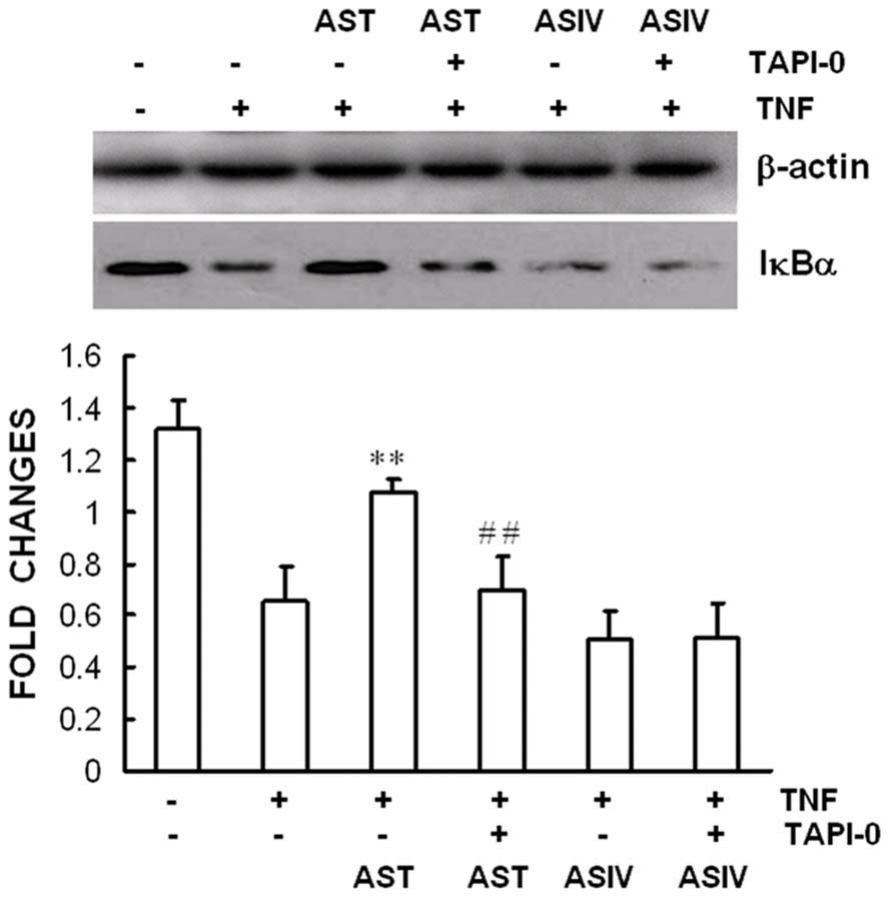
TAPI-0 reversed the inhibitory effect of AST on TNF-induced IκBα degradation. Confluent mAECs were pre-incubated with 4 µM of TAPI-0 for 30 min, then with 250 µg/mL of AST or ASIV for 2 h, and then they were treated with 30 ng/mL TNFα for 30 min. The expression of IκBα was measured by Western blot analysis. Data were representative of three independent experiments with determinations made in triplicate and shown as mean values+S.D. **P<0.01 compared with cells treated with TNF+CHX. ^##^ *P*<0.01 compared with cells treated with AST and then TNF+CHX.

Ectodomain shedding of cytokines and cytokine receptors plays a major role in the regulation of inflammatory processes. TACE (TNFα converting enzyme), also referred to as a disintegrin and metallopeptidase domain 17 (ADAM17), is a membrane-bound metalloprotease responsible for catalysing the shedding of the soluble TNF-α from membrane-bound pro-TNFα, and many other cell surface proteins including TNFR1, from cell surface. TNFR1 shedding has been shown to be a primary mechanism for the regulation of TNFα-mediated events. To determine if TACE played a role in the reduction of cell surface TNFR1 by AST, AECs were pre-incubated with 4 µM of TAPI-0, a synthetic inhibitor of TACE, for 30 min prior to TNFα stimulation and the TNFR1-mediated IκBα degradation was analyzed by Western blot. As shown in Figure 8, AST-induced inhibition of TNFR1-mediated IκBα degradation was significantly attenuated by the use of TAPI-0, strongly suggesting the involvement of TACE in the anti-inflammatory activity of AST. Further studies will be needed to provide evidence for AST-induced activation of TACE and to clarify the mechanisms of this action.

## Discussion

Saponins contained in *astragalus* have been investigated due to their cardioprotective effects, anti-inflammatory effects, and the potential to enhance longevity and lifespan. In this study we have made several novel observations and identified specific mechanisms through which saponins contained in astragalus inhibit TNFα-induced activation and cell damage in VECs. First we sought to compare the effects of AST and its major component ASIV on TNFα-induced up-regulation of CAMs expression and related NF-κB signaling pathway in murine AECs. Previous work by Zhang et al [11] demonstrated that ASIV, when used at the concentration of 100 µg/mL, significantly reduced LPS- and TNFα-induced expression of CAMs in HUVECs via inhibition of the nuclear translocation and phoshorylation of NF-κB-p65. Here we show that, similar to ASIV, AST also inhibited TNFα-induced expression of E-selectin and ICAM-1 genes (Figure 2). Additionally, TNFR1-mediated nuclear translocation and phophorylation of NF-κB-p65 were inhibited by both drugs (Figure 3, 4). This is quite conceivable as ASIV is thought to be the major active component in AST.

To better understand the mechanisms through which TNF-induced NF-κB activation was inhibited, we examined the upstream signaling event of TNFR1-mediated degradation of IκBα. To our surprise, the results showed that AST had a significant inhibitory effect on TNFR1-mediated IκBα degradation, while ASIV did not affect this signal (Figure 4). The results suggest that although both have a role in the regulation of TNFα-induced inflammatory responses in AECs, AST and ASIV act in different ways and use different mechanisms. This could be explained, at least partially, by the existence of other compositions, for example ASII or ASIII, in the total saponins (as shown in Figure 5). Previous work of Lee et al [2] also reported anti-inflammatory effects of other cycloartane-type saponin components in astragalus.

To further understand the differences in the inhibition of TNFR1-mediated signaling pathways by AST and ASIV, we examined their effects on TNFα-induced cell apoptosis. As shown in Figure 6, AST had a strong inhibitory effect on TNFα-induced activation of caspase-3 and protected against TNFα-induced apoptosis of AECs (Figure 6). In contrast, ASIV did not show significant protective effect on TNFα-induced apoptosis.

Given these results, we assumed that AST might inhibit TNFR1-mediated intracellular signaling pathways by preventing upstream initiation of the receptor signals. Our results showed direct evidence that AST, but not ASIV, reduced TNFR1 protein level on cell surface significantly while it did not affect TNFR1 expression in total cell lysates (Figure 7), suggesting the existence of a modulatory mechanism occurring at the level of the cell membrane. TNFR1 is the receptor type responsible for mediating most of the changes induced by TNFα. Presentation of TNFR1 on the cell surface is essential for the mediation of transmembrane signal transduction. Previous studies of ours and other researchers have shown that various stimuli, including endogenous G protein coupled receptor (GPCR) agonists such as histamine [17] and serotonin [18], chemotherapy drugs [19], TNFα itself [20], and even physiological mechanical stimulation [21] were able to dampen TNFα-caused inflammatory responses through reducing the amount of TNFR1 on cell surface. TACE, or ADAM17, is the major matrix metalloprotease responsible for ectodomain shedding of the TNF-α and TNFR1 from the cell surface, which is a primary mechanism for the regulation of TNFα-stimulated inflammatory processes. Our results that AST-induced inhibition of TNFR1-mediated IκBα degradation was significantly attenuated by pre-incubation of AECs with TAPI-0, a synthetic inhibitor of matrix metalloprotease and TACE, strongly suggests the involvement of TACE in the anti-inflammatory action of AST, but not ASIV (Figure 8). Further studies are required to prove AST-induced activation of TACE and to clarify the mechanisms underlying this action.

Altogether, the present study demonstrates the first evidence that ASIV is not superior to the less expensive AST in the attenuation of TNFα-induced inflammatory responses in AECs. The differences in the actions of these two drugs can be explained by components contained in AST other than ASIV. Furthermore, we would like to remark that, the results obtained with AECs were also be observed in other cell types, including mouse osteoblastic MC3T3 cells and monocytic RAW 264.7 cells (data not shown). Considering the role of TNFR1-mediated signaling pathways in the processes of osteoblastic and osteoclastic differentiation, AST may be one of the pharmaceutical ingredients responsible for the anti-osteoporotic effects of astragalus [22], [23].

In conclusion, the present study has shown the first evidence that, on the cellular level, ASIV was not superior to the less expensive AST, at least in the inhibition of TNFα-induced inflammatory responses in AECs that underlies the cardiovascular protective effect of astragalus. The inhibitory effect of AST was caused by the reduction of cell surface TNFR1 expression, which in turn might be induced by the upregulated activity of TACE. Further investigation is warranted to prove AST-induced activation of TACE and to elucidate the mechanisms underlying this action. Meanwhile, the mechanism through which ASIV inhibited TNFα-induced activation of NF-κB is another interesting question.
